# Remission Factors for Ustekinumab Treatment of Ulcerative Colitis: A Multicenter Retrospective Study of Real-World Data in Japan

**DOI:** 10.3390/biomedicines12051119

**Published:** 2024-05-17

**Authors:** Masashi Omori, Tomoyoshi Shibuya, Hirotaka Ishino, Yuka Fukuo, Rina Odakura, Masao Koma, Takafumi Maruyama, Kentaro Ito, Mayuko Haraikawa, Kei Nomura, Shintaro Yano, Osamu Nomura, Dai Ishikawa, Mariko Hojo, Taro Osada, Akihito Nagahara

**Affiliations:** 1 Department of Gastroenterology, Juntendo University School of Medicine, 2-1-1 Hongo, Bunkyo-ku, Tokyo 113-8421, Japan; ma-omori@juntendo.ac.jp (M.O.); r.odakura.sp@juntendo.ac.jp (R.O.); m-koma@juntendo.ac.jp (M.K.); t-maruyama@juntendo.ac.jp (T.M.); k.ito.wj@juntendo.ac.jp (K.I.); m.haraikawa.bk@juntendo.ac.jp (M.H.); ke-nomura@juntendo.ac.jp (K.N.); onomura@juntendo.ac.jp (O.N.); dai@juntendo.ac.jp (D.I.); mhojo@juntendo.ac.jp (M.H.); nagahara@juntendo.ac.jp (A.N.); 2 Department of Gastroenterology, Juntendo University Urayasu Hospital, 2-1-1 Tomioka, Urayasu-shi 279-0021, Japan; h.ishino.jl@juntendo.ac.jp (H.I.); shi-yano@juntendo.ac.jp (S.Y.); otaro@juntendo.ac.jp (T.O.); 3 Department of Gastroenterology, Juntendo University Nerima Hospital, 3-1-10 Takanodai, Nerima-ku, Tokyo 177-8521, Japan; yfukuo@juntendo.ac.jp

**Keywords:** lymphocyte-to-monocyte ratio, ulcerative colitis, ustekinumab, vedolizumab

## Abstract

Ustekinumab (UST) is an anti–IL-12/23p40 monoclonal antibody used to treat inflammatory bowel disease. The aim of this retrospective, multicenter study was to investigate the effectiveness of UST administration in achieving remission in patients with ulcerative colitis (UC) and to determine patient characteristics that influence its effectiveness. Of 88 UC patients who received UST from March 2020 to August 2023, 47 with traceable data and for whom 56 weeks had elapsed since the start of treatment received UST to induce remission. The remission rates at 8 weeks were 66% overall, 73.7% for Bio Naïve (never used biologics/JAK inhibitors), and 60.7% for Bio Failure (used biologics/JAK inhibitors) groups. Remission rates at 56 weeks were 70.2% overall, 73.7% for Bio Naïve, and 67.9% for Bio Failure groups. Ustekinumab showed good mid-to-long-term results in the induction of remission of UC in both Bio Naïve and Bio Failure groups. The group showing remission at 8 weeks had a significantly higher non-relapse or continuation rate (proportion of patients with no worsened symptoms necessitating surgery/drug change) at 56 weeks. Predictive factors for achieving remission after UST in UC were female gender, low body mass index, and low lymphocyte-to-monocyte ratio. Thus, UST is effective for moderate-to-severe UC.

## 1. Introduction

Ulcerative colitis (UC) is a chronic inflammatory intestinal disease that alternates between periods of remission and relapse, strongly and negatively affecting a patient’s quality of life [1]. In recent years, there has been a significant increase in the number of patients with inflammatory bowel disease (IBD). Globally, there were 6.8 million cases of IBD in 2017, with the age-standardized prevalence rate having increased from 79.5 per 100,000 people in 1990 to 84·3 per 100,000 in 2017 [2]. The age-standardized prevalence in 2017 was 464.5 per 100,000 people in the United States and 449.6 per 100,000 in the United Kingdom [2]. During the period from 1990 to 2019, IBD cases in Asia increased from approximately 760,000 to 2,000,000. In 2019, of four continents (Africa, America, Asia, and Europe), Asia demonstrated the highest absolute incidence and prevalence of IBD with approximately 150,000 incidences and 2 million prevalent cases [3]. The prevalence of IBD is also increasing in Japan, with recent reports putting the estimated number of patients at 220,000 for UC. The estimated annual prevalence rate of UC per 100,000 population, based on the mid-year population, of Japan in 2014 was 172.9 [4]. It has become a disease that is often seen in the daily practice of general internists.

Marked progress has been made recently in treating refractory UC; many biologics (Bio) and small-molecule compounds have been introduced such as anti–tumor necrosis factor antibodies [5,6,7], anti-integrin antibodies [8], and Janus kinase (JAK) inhibitors [9,10,11]. Ustekinumab (UST) is an interleukin-12/23 p40 antagonist that binds to a p40 subunit common to IL-12 and IL-23 and prevents their interaction with the β1 subunit of IL-12 and IL-23 receptor complexes. This prevents IL-12- and IL-23-mediated downstream signaling, gene activation, and cytokine production, which have a therapeutic effect on inflammatory diseases. Ustekinumab has recently become available for the treatment of patients with moderate-to-severe UC [12,13]. As described above, UST has a different formulation structure from that of conventional antibody drugs and is expected to demonstrate therapeutic efficacy in patients who have failed prior biologic drugs [14]. In recent years, reports have described the outcome of UST in UC [15,16,17]. However, how and in what order such treatments should be administered in each case have not yet been established.

Adverse events associated with the use of UST reported in more than five cases in the international phase III study (UNIFI study) include aggravation of UC, nasopharyngitis, and upper respiratory tract infection. The frequency of adverse events per 100 person-years was reported to not be significantly different between UST and placebo groups from the start of UST treatment to 156 weeks; however, real-world data are still scarce [18].

This retrospective multicenter study investigated outcomes in patients with UC undergoing UST treatment with regard to the induction of remission, the remission factors at 8 weeks, and any adverse events in an attempt to identify suitable patients for UST therapy. The purpose of this study is to aid clinicians in their future UC treatment of patients by exploring the optimal conditions for UST use in actual clinical practice.

## 2. Materials and Methods

### 2.1. Study Design

**Figure 1:**
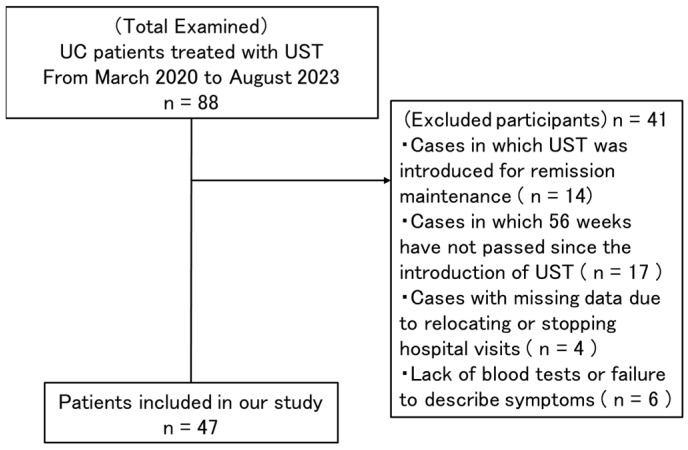
Study flow diagram. Eighty-eight patients with ulcerative colitis, treated with ustekinumab from March 2020 to August 2023, were enrolled. Of these, 47 patients were included in our study. UC, ulcerative colitis; UST, ustekinumab.

This multicenter retrospective study, conducted in Japan, included 88 UC patients with traceable data who received UST at Juntendo University School of Medicine, Juntendo University Nerima Hospital, and Juntendo University Urayasu Hospital from March 2020 to August 2023. Of these 88 patients, 14 patients, who received UST for remission maintenance with a Lichtiger index of four or less, were excluded. Of the remaining 74 patients for whom UST was administered for induction purposes, 17 were excluded because 56 weeks had not elapsed since the start of UST administration, 4 were excluded because they relocated or stopped visiting the hospital, and 6 were excluded because of missing blood test data or incomplete chart entries. Finally, 47 patients with traceable data who received UST for the induction of remission and for whom 56 weeks had elapsed since the start of treatment were included (Figure 1). In this study, an intention-to-treat analysis was performed, which included all patients who dropped out in the 56 weeks from the start of the study. Examination of medical records revealed data on diagnosis, clinical course, treatment, and laboratory parameters, including C-reactive protein (CRP), platelets, and differential leukocyte counts, which were de-identified and entered into an electronic database. The Juntendo University Hospital Ethics Committee (IRB no. E22-0124) reviewed and approved this retrospective study, which was conducted in accordance with standards for good clinical practice and the principles of the Declaration of Helsinki. Prior to the study, approval was obtained by the institutional review boards of all study centers.

### 2.2. Patients

A UC diagnosis was based on standardized criteria established by prior clinical assessment, endoscopy, and histology [19]. Specifically, UC was diagnosed when recurrent bloody or mucous stools, inflammation of the colonic mucosa extending from the rectum on endoscopic findings, or diffuse inflammatory cell infiltration and abnormal glandular arrangement characteristics of IBD on biopsy histology were observed [20].

Participants were divided into two groups: those who had never used biologics or JAK inhibitors before and those who had used them, defined as Bio Naïve and Bio Failure groups, respectively.

### 2.3. Treatment

Ustekinumab was administered intravenously with doses according to body weight (260 mg for body weight <55 kg, 390 mg for ≥55 kg to 85 kg, and 520 mg for ≥85 kg) in week 0 for the induction of remission and followed by a subcutaneous injection for the maintenance of remission. All patients were administered 90 mg of UST subcutaneously every 8 weeks; no patient was administered UST every 12 weeks.

### 2.4. Definition of Response

The Lichtiger index [21] evaluates disease activity scores eight items: (1) diarrhea (number of daily stools); (2) nocturnal diarrhea; (3) visible blood in the stool (% of movements); (4) fecal incontinence; (5) abdominal pain or cramping; (6) general well-being; (7) abdominal tenderness; and (8) need for antidiarrheal drugs. We defined remission as a Lichtiger index of 4 points or less and defined a clinical response as a decrease of at least 3 points and fewer than 10 points. The continuation rate was defined as the proportion of patients who did not require surgery or a change to another drug due to the worsening of symptoms.

### 2.5. Statistical Analysis

All data were analyzed by SPSS. Differences between groups were determined by a Mann–Whitney’s *U* test, Fisher’s test, and Chi-square test. The Kaplan–Meier method evaluated relapse-free survival. A significant difference was shown by *p* < 0.05.

## 3. Results

### 3.1. Patient Characteristics

Table 1 shows the characteristics of the 47 participants with UC (27 men, 20 women). At induction, their median age was 38 years, and median height and body mass index (BMI) were 163.4 cm and 21.3, respectively. The median Lichtiger index at the time of the introduction of UST was 9.7 points, and the patients mainly had moderate-to-severe disease. Of the 47 cases, 19 (40.4%) were in the Bio/JAK Naive group and 28 (59.6%) were in the Bio/JAK Failure group. Of the 28 Bio/JAK Failure patients, only one JAK Failure was a filgotinib failure. Treatment results and continuation rates at 8, 24, and 56 weeks were compared.

### 3.2. Therapeutic Effects of UST

**Figure 2:**
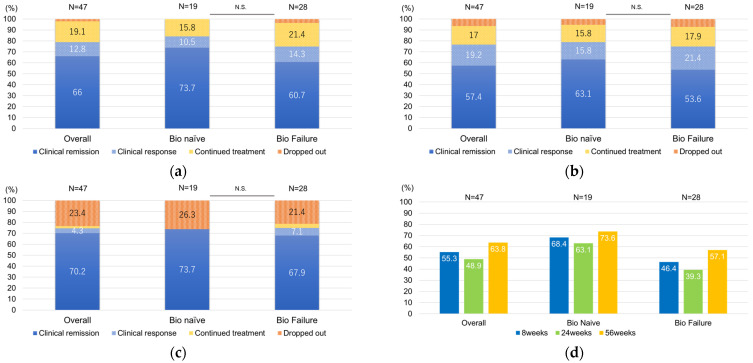
Clinical outcomes of UC patients treated with ustekinumab (**a**) at week 8, (**b**) week 24, and (**c**) week 56. The CS-free remission rate for overall, Bio Naïve, and Bio Failure groups over time (**d**). Bio Failure, used biologics/JAK inhibitors; Bio Naïve, never used biologics/JAK inhibitors; CS, corticosteroid.

**Figure 3:**
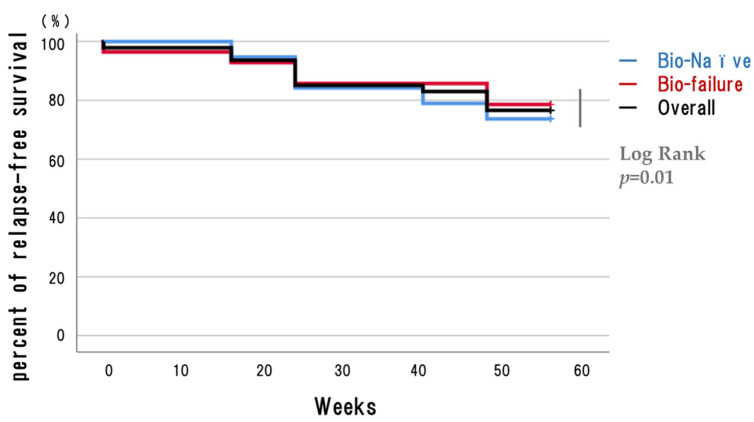
Survival (%). Kaplan–Meier plots for relapse-free survival of patients treated with ustekinumab up to 56 weeks after the initiation of medication. (Overall n = 47; Bio Naïve n = 19; Bio Failure n = 28). Bio Failure, used biologics/JAK inhibitors; Bio Naïve, never used biologics/JAK inhibitors.

The remission rates were as follows: at 8 weeks it was 66% overall, with 73.7% in the Bio Naïve group and 60.7% in the Bio Failure group (Figure 2a); at 24 weeks, it was 57.4% overall, with 63.1% in the Bio Naive group and 53.6% in the Bio Failure group (Figure 2b); and at 56 weeks, it was 70.2% overall, with 73.7% in the Bio Naive group and 67.9% in the Bio Failure group (Figure 2c). No statistically significant differences were observed between groups in remission or dropout rates at any time point. We also defined corticosteroid (CS)-free remission as remission without systemic CS use at that time point, regardless of initial steroid status, and analyzed results at 8, 24, and 56 weeks for the overall, Bio Naïve, and Bio Failure groups, respectively. The CS-free remission rate at 8 weeks was 55.3% overall, with 68.4% in the Bio Naïve group and 46.4% in the Bio Failure group; at 24 weeks, it was 48.9% overall, with 63.1% in the Bio Naïve group and 39.3% in the Bio Failure group, and at 56 weeks, it was 63.8% overall, with 73.6% in the Bio Naive group and 57.1% in the Bio Failure group. Notably, a trend was apparent toward higher remission rates in the Bio Naïve group at all time points (Figure 2d). A survival curve (Figure 3) was used to examine the cumulative non-relapse rate up to 56 weeks after induction, which included the percentage of patients not requiring surgery or conversion to another drug due to an exacerbation of symptoms. Persistence rates between the Bio Naïve and Bio Failure groups did not differ significantly (*p* = 0.134: Log Rank test).

### 3.3. Remission Factors and Continuation Rates

**Figure 4:**
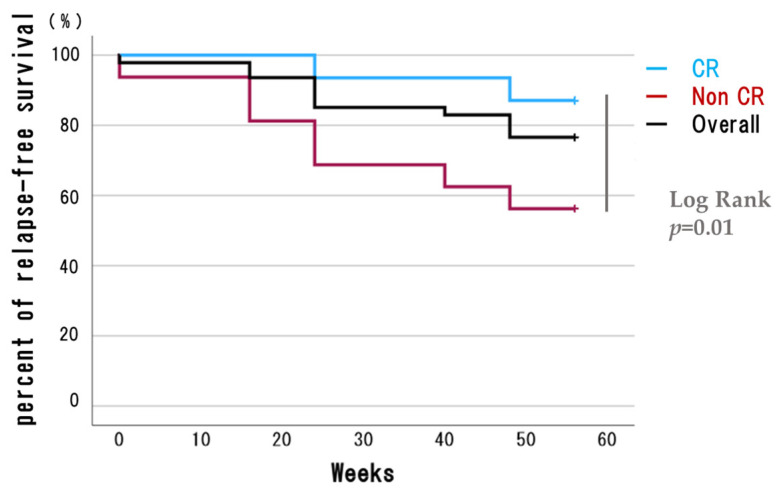
Clinical remission rate and relapse-free survival of patients treated with Ustekinumab. Kaplan–Meier plots up to 56 weeks after UST induction for the UC patient group that achieved clinical remission (CR) at 8 weeks and the group that did not (Non-CR). (Overall n = 47; CR n = 31; Non-CR n = 16). CR, clinical remission; UC, ulcerative colitis; UST, ustekinumab.

Patients in remission at 8 weeks and those not in remission were compared for the retention rate at 56 weeks. Of the total 47 patients, 31 patients who showed clinical remission (CR) at 8 weeks were in the CR group, and 16 patients who did not show CR were in the Non-CR group. Kaplan–Meier plots showed that the retention rate at 56 weeks was 27 (87%) in the CR group and 9 (56%) in the Non-CR group, with a significantly higher retention rate in the CR group by log-rank test (*p* = 0.01; Figure 4). These results suggest that achieving remission at 8 weeks is important for long-term maintenance. Therefore, factors at the time of induction were compared between two groups: those showing and not showing remission at 8 weeks. These included sex, age, BMI, initial dose, prior Bio/JAK use, concomitant azathioprine, concomitant prednisolone, CRP, Lichtiger index, Mayo endoscopic subscore, and platelet–lymphocyte, neutrophil–lymphocyte, and lymphocyte–monocyte ratios, respectively. In the group that achieved remission at 8 weeks, there were significantly more women, lower BMI, and more cases with an initial dose of 260 mg. Also, blood test results showed that the lymphocyte–monocyte ratio (LMR) was significantly lower in that group. No significant differences in severity of illness or concomitant medications were noted between groups (Table 2).

### 3.4. Examining the Effectiveness of UST Using the LMR

**Figure 5:**
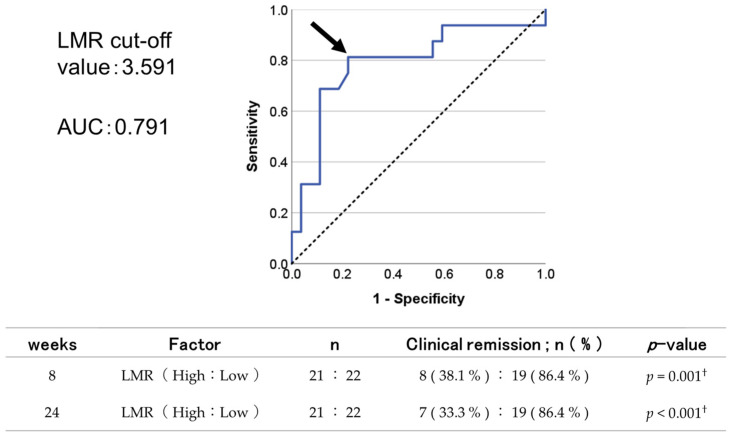
Receiver operating characteristic analysis of the LMR for predicting remission at 8 weeks in UC patients. Arrow is cut-off value obtained by Youden’s index. AUC, area under the curve; LMR, lymphocyte–monocyte ratio; UC, ulcerative colitis Weeks. Statistical analysis—^†^: Chi-square test.

The relationship between the LMR and UST obtained in a study of remission factors was examined by determining the cut-off value of the LMR from the receiver operating characteristic (ROC) curve and examining its usefulness. An ROC curve was drawn from logistic regression analysis of the relationship between the LMR and the presence of remission at 8 weeks to obtain the optimal LMR cut-off value (Figure 5). The area under the curve = 0.791 and a cut-off value of 3.591 were obtained using the Youden index. Therefore, we compared remission rates at 8 and 24 weeks in the UC patient group with an LMR ≥3.591 and the UC patient group with an LMR <3.591. The remission rate was found to be significantly higher in the group with the lower LMR (*p* = 0.001, *p* < 0.001; Pearson’s Chi-square test).

### 3.5. Effect of Vedolizumab Treatment History on UST

**Figure 6:**
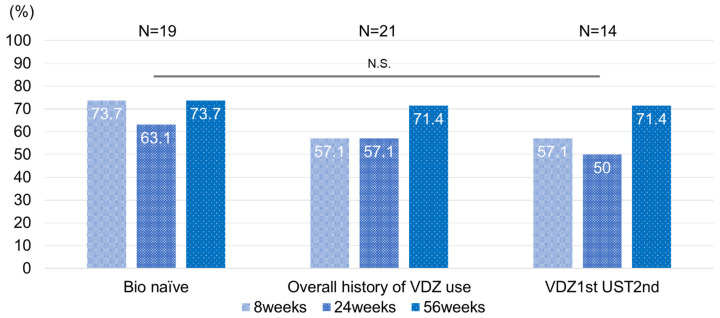
UST clinical remission rate by history of VDZ use in UC patients. Bio Naïve, never used biologics/JAK inhibitors; N.S., not significant; UC, ulcerative colitis; UST, ustekinumab; VDZ, vedolizumab.

We investigated the characteristics of 21 patients, who had used vedolizumab (VDZ) before UST, out of the 47 study participants. We compared the results at 8, 24, and 56 weeks in the Bio Naïve group, the entire group that had used VDZ before UST, and the group that had received VDZ as the first Biologics treatment and UST as the second Biologics treatment. The median number of VDZ doses for the 21 patients was 6, and the median time from the end of VDZ administration to the start of UST was 8 weeks (Table 3). We found that although the remission rate was slightly lower than that of the Bio Naïve group, more than 50% of the patients achieved remission at all time points, with no statistically significant difference (Figure 6).

### 3.6. Adverse Events

Two adverse events were reported in the observation period up to 56 weeks after the start of UST in the current study: The first case was a 54-year-old man who received nine doses of UST and reported the onset of facial nerve palsy. The patient was treated temporarily with oral steroids. His symptoms subsequently resolved, and he was able to continue UST as planned. However, the diagnosis and treatment of the facial nerve palsy were undertaken at another hospital, and details regarding the dose and duration of steroid administration are unknown. The second case was a 38-year-old woman who had been treated with UST for the induction of remission. However, after four doses, she developed arthralgia, which made it difficult to continue treatment, and she was switched to adalimumab. At that time, no exacerbation of gastrointestinal symptoms was observed. No other potentially fatal adverse events were observed.

## 4. Discussion

The overall remission rate of UST in our 47 patients with UC at 8 weeks was 66%, which is comparable to the overall clinical response rate of 61.8% at 8 weeks in the data of the UNIFI study, an international phase III study of UST [13]. Both the Bio Naïve and Bio Failure groups also showed better outcomes than the UNIFI study [13]. In our study, UST treatment showed good retention rates up to 56 weeks, regardless of whether or not a biologic agent was previously used, and no statistically significant differences were observed. Other real-world data showed similar results. Ustekinumab real-world data from a study of 108 patients in Spain showed CR rates of 59%, 57%, and 69% at 8, 24, and 52 weeks, respectively [22]. However, a report from Switzerland described introducing UST for 133 patients, with a continuation rate of 86% at 16 weeks and a remission rate of 17% at 16 weeks. These results were somewhat lower than the results of the UNIFI study and our study. The authors, Thunberg et al., attributed the lower remission rate to the difference in the number of Bio Naïve cases, which was 49% in the UNIFI study and only 2% in the Swiss study [23]. In our study, the figure for Bio Naïve cases was 40%, and the conditions were more similar to the UNIFI study compared to the Swiss study. Therefore, a trend may exist toward higher remission rates in Bio Naïve cases, although this difference was not significant in our study.

In terms of adverse events, 2 of 47 patients in this study experienced adverse events; one patient had to be changed to another treatment. Serious adverse events at 11.5 per 100 patient-years were reported in the first year of the UNIFI study. Compared to that result, the number of adverse events in this study was low. Furthermore, the UNIFI trial reported data through the third year after the start of UST, with 7.5 serious adverse events per 100 patient-years at the third year, with no increase in adverse events over time [18]. This may be considered significant data for the long-term continuation of UST.

Regarding remission factors in the use of UST, significantly more women and lower BMI values were characteristic of the group that achieved remission at 8 weeks after induction than in the group that did not achieve remission. In addition, the number of patients weighing ≤55 kg who were eligible for the initial dose of 260 mg was significantly higher in the group that achieved remission at 8 weeks. Four cases were administered a first dose of 390 mg for some reason even though the patient weighed <55 kg at the time of administration. No bias was observed toward females, with two males and two females among the four who received the higher dose. The dose of UST was constant at 90 mg for the second and subsequent subcutaneous injections regardless of body weight. However, since this study only examined the effect of the first intravenous dose, it is unlikely that a significant difference exists in concentration per body weight. This trend was also reported in the UNIFI study. The CR rate at 8 weeks by patient background was 13.8% for males and 18.1% for females in 322 patients in the 6 mg/kg group. By weight, 24.1% of patients in a 6 mg/kg group weighed 55 kg or less; 11.9% in those weighing more than 55 kg and less than 88 kg, and 18.3% in those weighing more than 85 kg, with the lightest-weight group showing the highest remission rate. It is interesting to note that the same trend as in the present study was observed, although the difference was not significant. Although the small number of patients in this study may have contributed to the significant difference, further analysis of the effects of gender and body weight on immune response is needed. The reasons for such results also require further investigation, but findings suggest that remission of UC is more likely to be achieved with UST in petite women.

The LMR is an indicator of an inflammatory response and has recently been reported to be associated with prognosis in various malignancies. In reports of many carcinomas, such as esophageal cancer [24], gastric cancer [25], and colorectal cancer [26], a low preoperative LMR value is a poor prognostic factor. However, although few reports exist on the relationship between the LMR and UC, the LMR is expected to be low during the active inflammatory phase because the lymphocyte proportion of leukocytes decreases and the monocyte proportion increases. In fact, UC activity was high, with a sensitivity of 76%, when the LMR was lower than the cut-off value of 3.1 [27]. It has also been reported that the LMR can predict treatment failure within 3 months in patients receiving advanced therapy for active UC [28]. Based on a cut-off value of 3.591 obtained in Study 3.4, the Lichtiger index was analyzed for high and low LMR groups. Although a tendency existed for this index to be slightly higher in the low LMR group, no significant difference was noted in severity of illness according to the LMR (*p* = 0.420). No report exists of the use of the LMR to examine remission factors related to UST for UC. In our study, the remission rate at 8 weeks after UST induction was higher in patients with a lower LMR, a result seemingly contradicting previous reports. Interleukin-12 is an inflammation-inducing cytokine, produced by monocytes and monocyte-differentiated macrophages and dendritic cells, that stimulates naive T cells and natural killer cells, induces the production of interferon-γ, and promotes helper T 1 cell differentiation [29]. Interleukin-23 is also produced by monocytes, monocyte-differentiated macrophages, and dendritic cells; is a heterodimer composed of IL-12/23p40, which is common to IL-12 and IL-23p19, and that is unique to IL-23 [30]. Therefore, predominantly elevated monocytes, as well as IL-12 and IL-23 levels, are thought to have a major role in the remission rate after UST induction since UST inhibits these. The LMR is an inexpensive and simple marker that can be calculated from blood tests performed in routine practice; if the LMR can be calculated to predict UC remission in advance, it may be an important indicator for UST-based UC treatment.

Currently no consensus exists on the outcome of the use of UST for UC patients with VDZ failure. Although our results showed a slight decrease in efficacy compared to the non-failure group for biologic agents, treatment outcome did not differ significantly based on a history of VDZ use, regardless of whether or not other drugs were used before or after VDZ. These results suggest that UST is sufficiently useful for UC patients with a history of VDZ use. In recent years, many therapeutic agents have become available for UC, and although each has a different mechanism of action, much overlap exists in the cases that they target. In order to utilize these drugs as effectively as possible, it is important to know which drugs are more effective for which patients. If we can predict remission in advance by exploring the remission factors of each drug, an increasing success rate of treatment is foreseeable, which is highly significant. In addition, it is very important to know in which order the next drug should be selected when treatment fails. Patients with IBD need to continue treatment throughout their lives; it is important to consider the sequence of treatments in order to obtain long-lasting effects over a long lifetime of treatment.

Our study has several limitations. First, the number of participants was small; therefore, case bias cannot be ruled out. Second, because of this, multivariate analysis could not be performed, and the effects of confounding factors could not be excluded. Third, UST administration was given only once every 8 weeks; results may differ in the case of administrations every 12 weeks.

Consequently, we would like to analyze various confounding factors by accumulating more cases and conducting multivariate analyses to identify more essential remission factors. In addition, we would like to clarify the appropriate order of UST use by analyzing the results of UST use in patients with a history of other drug use, as well as VDZ. We believe that an analysis of patients who receive UST at 12-week intervals will provide more clinically useful data.

## 5. Conclusions

In conclusion, UST is effective in real-life clinical practice for moderate-to-severe UC regardless of the prior use of Bio or JAK inhibitors. A history of VDZ use has no significant effect on the efficacy of UST treatment. The patient group showing remission at 8 weeks had a significantly higher continuation rate at 56 weeks; being female with a small build and having a low LMR at the time of UST induction were found to be predictive for remission. In particular, a low LMR may be an early-to-mid-term remission indicator. The results of this study will provide important information for clinicians in predicting remission when UST is introduced to patients with moderate-to-severe UC.

## Figures and Tables

**Table 1:** UC patient characteristics.

		(n = 47)	
Age, median ± SD (years) [range]		38.0 ± 15.0 [20–76]	
Sex, male:female		27:20	
Height, median ± SD (cm) [range]		163.4 ± 7.9 [144–178.5]	
BMI, median ± SD [range]		21.3 ± 3.2 [14.5–28.7]	
Disease duration, median ± SD (year) [range]		5 ± 8.9 [1–34]	
Clinical Activity (Lichtiger index), median ± SD [range]		9.7 ± 3.3 [5–16]	
Mayo Endoscopic Subscore, mean ± SD [range]		2 ± 0.7 [0–3]	
Location of colitis n (%)	Pancolitis	35 (74.5%)	
	Left-sided colitis	8 (17.0%)	
	Proctitis	4 (8.5%)	
Initial dose, 260 mg: 390 mg		17:30	
Bio/JAK naïve, n (%)		19 (40.4%)	
Bio/JAK failure, n (%)	1	28 (59.6%)	17 (36.2%)
	2		10 (21.3%)
	3		1 (2.1%)
Concomitant drug, n (%)	5-Aminosalicylate	42 (89.4%)	
	Prednisolone	9 (19.1%)	
	Azathioprine	16 (34%)	

BMI, body mass index; SD, standard deviation.

**Table 2:** Factors predicting the clinical remission of UC in patients treated with UST Statistical analysis—^†^: Chi-square test; ^††^: Fisher’s test; ^§^: Mann–Whitney’s *U* test.

Factor	n	Clinical Remission; n (%)	*p*-Value
Sex (male: female)	27:20	12 (44.4%):19 (95%)	*p* < 0.001 ^†^
Age [range: 20–76]	-	-	*p* = 0.645 ^§^
BMI [range: 14.5–28.7]	-	-	*p* = 0.024 ^§^
Initial dose (260 mg:390 mg)	17:30	15 (88.2%):16 (53.3%)	*p* = 0.015 ^†^
Prior Bio/JAK use (YES:NO)	28:19	17 (60.7%):14 (73.7%)	*p* = 0.357 ^†^
Concomitant Azathioprine (YES:NO)	16:31	10 (62.5%):21 (67.7%)	*p* = 0.719 ^†^
Concomitant Prednisolone (YES:NO)	9:38	6 (66.7%):25 (65.8%)	*p* = 1.000 ^††^
CRP [range: 0.02–14.95]	-	-	*p* = 0.704 ^§^
Lichtiger index [range: 5–16]	-	-	*p* = 0.874 ^§^
Mayo Endoscopic Subscore [range: 0–3]	-	-	*p* = 0.899 ^§^
Platelet–lymphocyte ratio [range: 87.1–682.3]	-	-	*p* = 0.393 ^§^
Neutrophil–lymphocyte ratio [range: 0.82–15.1]	-	-	*p* = 0.183 ^§^
Lymphocyte–monocyte ratio [range: 0.5–18.9]	-	-	*p* = 0.002 ^§^

BMI, body mass index; CRP, C-reactive protein; UC, ulcerative colitis; UST, ustekinumab.

**Table 3:** Summary of information on UC patients with a history of VDZ use.

	Frequency of VDZ Administration	Period from VDZ Discontinuation to UST Start (Week)	VDZ Primary Invalidity or Loss of Response	Course of Treatment up to the Start of UST
1	5	8	loss of response	VDZ→UST
2	10	7	loss of response	VDZ→UST
3	1	1	primary invalidity	VDZ→UST
4	4	24	loss of response	VDZ→UST
5	6	49	primary invalidity	VDZ→IFX→UST
6	9	8	loss of response	GLM→VDZ→UST
7	10	8	loss of response	GLM→VDZ→UST
8	6	7	loss of response	VDZ→UST
9	5	8	primary invalidity	VDZ→UST
10	5	5	primary invalidity	IFX→VDZ→UST
11	1	55	primary invalidity	VDZ→ADA→UST
12	7	9	primary invalidity	VDZ→UST
13	3	6	loss of response	VDZ→UST
14	6	1	loss of response	VDZ→UST
15	3	8	primary invalidity	VDZ→UST
16	16	5	loss of response	VDZ→UST
17	2	7	primary invalidity	ADA→VDZ→TOF→UST
18	24	8	loss of response	ADA→VDZ→UST
19	9	8	loss of response	VDZ→UST
20	14	4	loss of response	VDZ→UST
21	3	4	primary invalidity	VDZ→UST

ADA, adalimumab; IFX, infliximab; GLM, golimumab; TOF, tofacitinib; UST, ustekinumab; VDZ, vedolizumab.

## Data Availability

The data presented in this study are available on request from the corresponding author.
